# Obesity and Risk of Bladder Cancer: A Dose-Response Meta-Analysis of 15 Cohort Studies

**DOI:** 10.1371/journal.pone.0119313

**Published:** 2015-03-24

**Authors:** Jiang-Wei Sun, Long-Gang Zhao, Yang Yang, Xiao Ma, Ying-Ying Wang, Yong-Bing Xiang

**Affiliations:** 1 State Key Laboratory of Oncogene and Related Genes, Shanghai Cancer Institute, Renji Hospital, Shanghai Jiaotong University School of Medicine, Shanghai, China; 2 Department of Epidemiology, Shanghai Cancer Institute, Renji Hospital, Shanghai Jiaotong University School of Medicine, Shanghai, China; Louisiana State University Health Sciences center, UNITED STATES

## Abstract

**Background:**

Epidemiological studies have reported inconsistent association between obesity and risk of bladder cancer, and the dose-response relationship between them has not been clearly defined.

**Methods:**

We carried out a meta-analysis to summarize available evidence from epidemiological studies on this point. Relevant articles were identified by searching the PubMed and Web of Science databases through September 30, 2014. We pooled the relative risks from individual studies using random-effect model, and the dose—response relationship was estimated by using restricted cubic spline model.

**Results:**

Fifteen cohort studies with 38,072 bladder cancer cases among 14,201,500 participants were included. Compared to normal weight, the pooled relative risks and corresponding 95% confidence intervals of bladder cancer were 1.07(1.01-1.14) and 1.10(1.06-1.14) for preobese and obesity, with moderate (*I^2^* = 37.6%, *P* = 0.029) and low (*I^2^* = 15.5%, *P* = 0.241) heterogeneities between studies, respectively. In a dose-response meta-analysis, body mass index (BMI) was associated with bladder cancer risk in a linear fashion (*P*_non-linearity_ = 0.467) and the risk increased by 4.2% for each 5 kg/m^2^ increase. No significant publication bias was found (*P* = 0.912 for Begg’s test, *P* = 0.712 for Egger’s test).

**Conclusions:**

Findings from this dose-response meta-analysis suggest obesity is associated with linear-increased risk of bladder cancer.

## Introduction

An estimated 429,793 new cases and 165,068 deaths from bladder cancer occurred in 2012 worldwide [1]. Established risk factors for bladder cancer include cigarette smoking, schistosomal infection, occupational exposure to specific carcinogens such as aromatic amines, drinking tap water with arsenic, and familial history of bladder cancer [2]. In the past decades, extensive evidence suggested potential associations between obesity and many cancers [3–10], it is convincing for obesity as a risk factor for cancers of esophagus [4], pancreas [5], colon and rectum [6], endometrium [7,8], kidney [9],and postmenopausal breast[10]. However, epidemiological studies have reported inconsistent associations between body mass index (BMI; weight in kilograms divided by height squared in meters) and bladder cancer risk [11–25]. When results were combined in a meta-analysis published in 2013 [26], obesity was associated with a 10% increase in risk of bladder cancer. In subgroup analysis, however, this previous review had not analyzed the influence of potential confounders (e g: physical activity, alcohol consumption and family history of cancer) on the association between obesity and bladder cancer risk. Meanwhile, it did not examine the exact shape of the dose-response relationship between BMI and bladder cancer risk.

Therefore, we conducted a systematic review and a dose-response meta-analysis of published cohort studies to update and expand the previous meta-analysis. Further, we assessed the influence of preobese and obesity on bladder cancer risk separately.

## Materials and Methods

### Literature search

Articles were identified by using the PubMed and Web of Science databases through September 30, 2014, with the terms: “body mass index”, “BMI”, “overweight”, or “obesity”, together with “bladder cancer” or “bladder neoplasm”. No restrictions were imposed. In addition, the reference lists of the retrieved articles were screened for qualifying studies.

### Eligibility criteria

Two authors (J-WS and L-GZ) independently confirmed the eligibility of studies based on the selection criteria. To be included, the study had to use cohort design, report the RRs/HRs with corresponding 95% confidence intervals (CIs) or reported data to calculate these. Studies were excluded if the study evaluated association of BMI and bladder cancer risk with standardized incidence ratios. When multiple publications from the same study were available, we used either the most recent publication or the publication with most-applicable information and the largest number of cases. Discrepancies between the two reviewers were solved by discussion.

### Data extraction

Two investigators (J-WS and L-GZ) extracted the following data from each included study: the first author’s last name, publication year, country or region, study name, sex, age, sample size (number of cases, participants or person-years), length of follow up, assessment of BMI (measured or self-reported), risk estimates and 95% confidence intervals, covariates adjusted for in the multivariable analysis. For studies that reported several multivariable-adjusted RRs, we selected the risk estimates that adjusted for most potential confounders. The BMI (kg/m^2^) in adults was classified as follows: normal weight, 18.50–24.99; preobese, 25.00–29.99; obesity, ≥30 [27].

### Statistical analysis

Summary RR estimates with their corresponding 95% confidence intervals were derived by using a random-effect model [28] based on statistically significant heterogeneity. Heterogeneity was assessed using *Q* and *I* ^*2*^-statistics (*P*<0.10 or *I* ^2^>50% was used as a threshold indicating statistically significant heterogeneity) [29]. If a study reported risk estimates for men and women separately, we included both risk estimates in the meta-analysis because they were based on independent samples. Subgroup analyses were conducted across a number of key study characteristics to investigate the effect of potential confounders, such as gender (men, women), study location (Asia, Europe, North America), assessment of BMI (measured, self-reported), duration of follow up (<10 years, ≥10years), adjustment for smoking (yes, no), physical activity (yes, no), alcohol consumption (yes, no), and family history of cancer (yes, no). Publication bias was evaluated by using Egger’s test [30] and Begg’s test [31].

For dose-response analysis, the numbers of outcomes and persons/person-years for at least three BMI categories, and means or medians of the categories, or if not reported in the studies, the estimated midpoints of the categories had to be available. When the extreme BMI categories were open-ended, we used the width of the adjacent close-ended category to estimate the midpoints [32]. Because the reference of exposure are different or not zero across studies, centered dose levels (each original non-reference dose minus the reference dose within a study) was used for summarizing dose-response relation[33]. We performed a 2-stage random-effects dose-response meta-analysis to examine the potential trend between BMI and bladder cancer risk [34, 35]. This was applied by modeling BMI using restricted cubic spline model with three knots (2 spline transformations) chosen at the 10%, 50% and 90% percentiles of the distribution [36]. In the first stage, a restricted cubic spline model was fitted by using generalized least-squares regression by taking into account the correlation within each set of specific relative risks to estimate the 2 study-level coefficients and the within-study covariance matrix[34,35]. In the second stage, we derived the overall estimates with random effects by pooling the study-specific coefficient estimates and variance/covariance matrices that had been obtained in the first stage [37]. Consequently, the combined estimates of the HRs for the BMI groups take into account both the variation within studies and the variation between studies. We calculated a *P*-value for nonlinearity by testing the hypothesis that the coefficient of the second spline was different from zero [34]. For the study included in the dose-response analysis, we estimated a RR and corresponding 95% confidence intervals for a 5 kg/m^2^ increase in BMI.

To assess whether the summary results are robust, we conducted two sensitivity analyses: first, omitting one study at a time and examining the influence of each individual study on the overall relative risk; second, using other percentiles (5, 35, 65 and 95%) of the distribution as knots for dose-response meta-analysis.

All statistical analyses were performed with Stata, version12 (Stata Corp,College Station, TX). *P* values of less than 0.05 were considered statistically significant.

## Results

### Study characteristics

**Fig 1:**
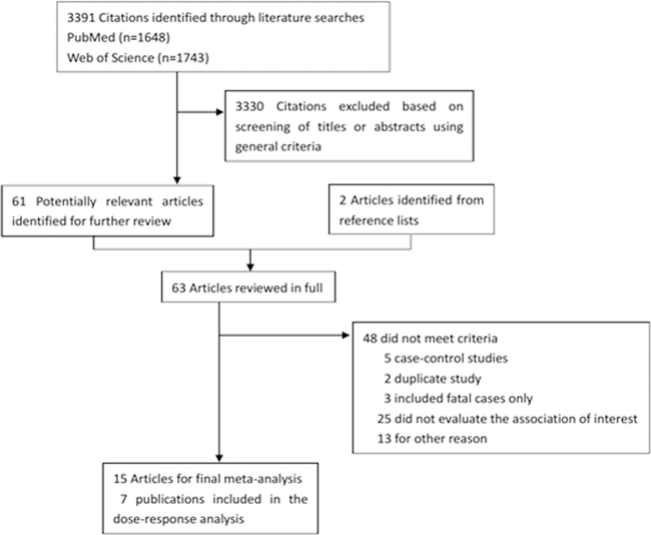
Flow diagram of the study selection for the meta-analysis.

A flowchart of the identification of relevant studies is shown in Fig. 1. Fifteen cohort studies with 38,072 bladder cancer cases among 14,201,500 participants were included in our meta-analysis. Six studies conducted in North American, 5 in European, 2 in Asian, 1 in Australia, and 1 was the Metabolic syndrome and Cancer project(Me-Can) with subjects recruited from Norway, Sweden, and Austria. Seven studies [11–15,18,23] were included in the dose-response analysis. Few studies controlled for physical activity[12,14,19] and alcohol consumption[12,14,19,22], but 12 studies [11–15,17–21,23,24]controlled for cigarette smoking. Characteristics of the included studies in this meta-analysis were shown in S1 Table.

### Categorical meta-analysis

**Fig 2:**
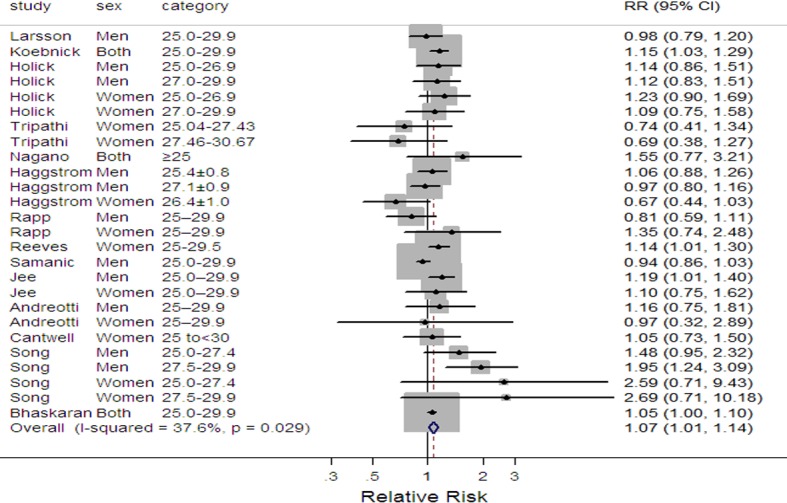
Forest plot of preobese and risk of bladder cancer. Squares indicate study-specific relative risks (size of the square reflects the study-specific statistical weight, i.e., the inverse of the variance); horizontal lines represent 95% CIs; the diamond indicates the summary relative risk estimate with its 95% CI. CI, confidence interval.

**Fig 3:**
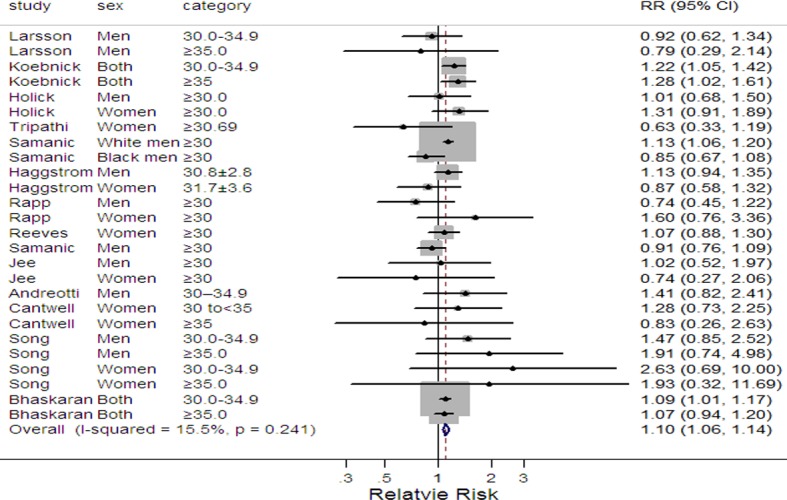
Forest plot of obesity and risk of bladder cancer. Squares indicate study-specific relative risks (size of the square reflects the study-specific statistical weight, i.e., the inverse of the variance); horizontal lines represent 95% CIs; the diamond indicates the summary relative risk estimate with its 95% CI. CI, confidence interval.

Compared to normal weight, the pooled relative risks and the corresponding 95% confidence intervals of bladder cancer were 1.07(1.01–1.14) and 1.10(1.06–1.14) for preobese (Fig. 2) and obesity (Fig. 3), with moderate (*I* ^2^ = 37.6%, *P* = 0.029) and low (*I* ^2^ = 15.5%, *P* = 0.241) heterogeneities between studies, respectively. The summary RR for each 5 kg/m^2^ increase in BMI was 1.04 (95%CI 1.01–1.07, *I* ^2^ = 32.1%, *P* = 0.172) (S1 Fig.).

### Dose-response meta-analysis

**Fig 4:**
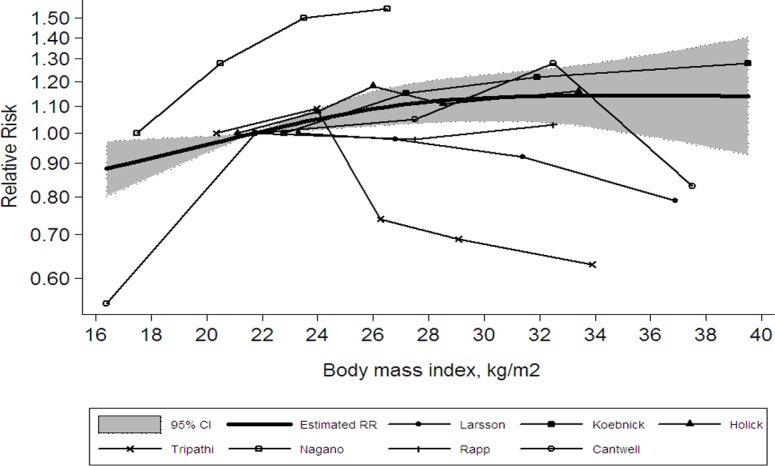
Dose-response relationships between body mass index and the relative risk of bladder cancer. Shaded area represents 95% confidence limits for fitted curve. *P* = 0.467 for non-linearity, which indicates no curvilinear association.

By using restricted cubic spline model, the result revealed a linear relationship between BMI and risk of bladder cancer (*P*_non-linearity_ = 0.467). When linear models were fitted, it showed an increased bladder cancer risk of 4.2% for each 5 kg/m^2^ increase in BMI (Fig. 4).

### Subgroup analysis

In the subgroup analysis (Table 1), we performed stratified analysis across a number of key study characteristics. Stronger associations between BMI and bladder cancer risk were found if BMI was assessed by self-reported, and if the average age of participants was greater than 50 years old. No significant effect differences were observed for duration of follow-up and for the gender of participants. The summary RR was slightly higher in Asian studies than that in North American or European studies for preobese; but for obesity, the summary RR for North American was slightly higher than that for Asian or European studies. When we restricted the meta-analysis to studies that had adjusted for potential confounders, a prominent association was observed. For example, stronger associations were found in studies adjusting for physical activity, alcohol consumption, or family history of cancer then studies that did not adjust for such confounders. For preobese, some evidence of heterogeneity was found in men (*I* ^2^ = 59.0%), in studies located in Europe (*I* ^2^ = 66.4%), in studies which BMI was measured (*I* ^2^ = 58.9%).

**Table 1:** Subgroup analysis of body mass index and risk of bladder cancer.

Stratification criterion	Preobese					Obesity			
	No. of studies		RR（95%CI）	I^2^(%)	*P*-value*	No. of studies	RR（95%CI）	I^2^(%)	*P*-value*
Gender									
Men	9	1.09(0.99–1.20)		59.0	0.005	10	1.10(1.05–1.16)	30.9	0.129
Women	10	1.07(0.98–1.17)		27.4	0.168	9	1.13(1.00–1.29)	3.4	0.411
Age									
<50	9	1.06(0.98–1.15)		44.2	0.023	8	1.08(1.02–1.14)	9.2	0.350
≥50	6	1.11(1.03–1.19)		31.0	0.170	7	1.12(1.06–1.18)	16.0	0.283
Study location									
Asia	2	1.19(1.03–1.38)		0.0	0.709	1	0.93(0.53–1.62)	N/A	N/A
Europe	5	1.09(0.98–1.22)		66.4	0.004	5	1.07(1.01–1.13)	1.8	0.422
North America	5	1.12(1.03–1.22)		0.0	0.820	6	1.13(1.07–1.19)	29.9	0.170
Assessment of BMI									
Measured	6	1.10(0.98–1.23)		58.9	0.007	7	1.09(1.04–1.14)	24.0	0.189
Self-reported	7	1.12(1.04–1.19)		0.0	0.760	6	1.15(1.05–1.26)	7.2	0.375
Duration of follow up									
<10years	5	1.07(1.02–1.11)		31.8	0.197	5	1.10(1.05–1.16)	5.7	0.388
≥10years	9	1.08(0.99–1.19)		15.6	0.275	9	1.10(1.04–1.15)	24.0	0.176
Adjustment factors									
Smoking									
Yes	13	1.07(0.99–1.16)		46.1	0.007	12	1.10(1.03–1.18)	8.8	0.339
No	2	1.05(1.00–1.10)		0.0	0.888	2	1.10(1.05–1.15)	45.0	0.142
Physical activity									
Yes	5	1.15(0.97–1.36)		52.7	0.025	4	1.20(1.08–1.33)	2.7	0.414
No	10	1.03(1.00–1.07)		15.5	0.272	10	1.09(1.04–1.13)	6.9	0.373
Alcohol									
Yes	4	1.13(1.04–1.22)		0.0	0.451	4	1.17(1.06–1.30)	30.2	0.220
No	10	1.06(0.99–1.14)		41.3	0.029	10	1.09(1.05–1.13)	9.0	0.342
Family history of cancer									
Yes	1	1.15(1.03–1.29)		N/A	N/A	1	1.24(1.09–1.40)	N/A	N/A
No	13	1.06(1.00–1.13)		36.1	0.038	13	1.09(1.02–1.13)	10.4	0.317

BMI, body mass index; RR, relative risk; CI, confidence interval; N/A, not available.

*P-value for heterogeneity within each subgroup

### Sensitivity analysis

In a sensitivity analysis, we sequentially omitted one study at a time from the meta-analysis to examine whether the main results were influenced by a particular study. The summary RRs for obesity ranged from a low of 1.08(95%CI 1.03–1.13) after omitting the study by Samanic et al.[16] to a high of 1.11(95%CI 1.07–1.15) after omitting the study by Samanic et al. [20], which indicated the association between obesity and bladder cancer was not influenced by a single study. The summary RRs for preobese ranged from a low of 1.05 (95%CI 0.99–1.12) after omitting the study by Koebnick et al.[12] to a high of 1.08(95%CI 1.02–1.15) after omitting the study by Samanic et al.[20]. Meanwhile, we restricted the analyses to the studies that provided estimates with exclusion of early deaths to minimize the effect of reverse causality. The results were not substantially changed (summary RR = 1.06 (95%CI 0.98–1.14, *I* ^*2*^ = 29.9%, *P* = 0.179) for preobese, and 1.12 (95%CI 1.06–1.18, *I* ^*2*^ = 13.03%, *P* = 0.222) for obesity). The dose-response meta-analysis used four knots at percentiles 5, 35, 65 and 95% of the distribution acquired similar results with the dose-response meta-analysis with three knots (*P*_non-linearity_ = 0.376).

### Publication bias

The *P* values of Begg’s test and Egger’s test were 0.912 and 0.712, respectively. No significant publication bias was detected in our meta-analysis.

## Discussion

The findings of this meta-analysis of 15 cohort studies indicate that preobese has a statistically significant 7% increased risk of bladder cancer, while obesity increases the risk of bladder cancer by approximately 10%. The dose-response meta-analysis suggests a linear association between BMI and bladder cancer, and showed each 5 kg/m^2^ increment of BMI corresponded to a 4.2% increase in risk of bladder cancer.

Compared to the previous quantitative review by Qin et al.[26], our meta-analysis was also explore the association between preobese and bladder cancer risk, and the dose-response association between BMI and risk of bladder cancer was also investigated. In this updated meta-analysis, although the associations between BMI and bladder cancer risk were not influenced by the duration of follow-up, we cannot rule out the possibility that reverse causality could lead to biased results. However, our finding of robust results between BMI and bladder cancer risk from sensitivity analyses (restricted analysis to studies that excluding early deaths) argues against the notion that reverse causality explained our findings. Sufficiently long follow-up times are important because most cancers usually have a latent period of years or even decades [38]. Most studies included in our analyses had average follow-up times exceeding 10 years, including three studies with median follow-up times of over 15years. If a true association between obesity and bladder cancer existed, the follow-up periods in our analyses should have been long enough to detect such an association.

Although smoking is an established risk factor for bladder cancer [39] and many studies have indicated that smoker’s BMI is lower [40, 41], adjustment for smoking did not affect the association of obesity to bladder cancer in our meta-analysis. This suggests that the biological mechanisms through which obesity may increase bladder cancer risk are not mediated by the influence of smoking, which is supported by several cohort studies [11–14, 21]. Besides smoking, the influences of other potential confounders such as physical activity, alcohol consumption, and family history of cancer were also estimated in subgroup analysis. We observed stronger associations in studies that adjusted for those confounders than studies that did not adjusted for those confounders. Because self-reported BMI could lead to misclassification bias by underestimating the true BMI, studies using self-reported BMI showed a stronger association than those using measured BMI. However, high validity has been observed for self-reported and measured height and weight [42, 43]. Thus, this underestimation should not affect the overall qualitative inference that increasing BMI is associated with high risk of bladder cancer. Furthermore, in our meta-analysis, the results were similar when studies were stratified by the method of BMI assessment.

The biological mechanisms driving the positive association between obesity and bladder cancer are not well understood. Obesity is associated with elevated production of insulin, which is a mitogenic factor that may also enhance tumor growth by increasing free insulin-like growth factor-I[44], which in turn modify cell proliferation, apoptosis and angiogenesis [45], and plays a role in bladder cancer[46, 47]. In addition, obesity may also induce chronic low-grade inflammation resulting in an alteration of local and systemic levels of cytokines (e.g., interleukin-6, C-reactive protein) and adipokines (e.g., leptin, adiponectin) [48], which may play a role in bladder carcinogenesis to bladder cancer mortality [49, 50]. Each of these factors, although, may play an independent role in carcinogenesis, it is more likely that those factors act cooperatively in the carcinogenic process.

Our meta-analysis has several strengths. First, we restricted our analysis to prospective studies and excluded traditional case-control studies, which would eliminate the influences causing by recall bias. Second, to our knowledge for the first time in a meta-analysis, dose-response analysis was performed to better describe the relationship between BMI and bladder cancer. Third, a large number of cohort studies with relatively long follow-up and large sample size enable us to conduct a wide range of informative analysis in various subgroups of populations. The results were generally robust and were not influenced by a particular study.

However, our meta-analysis also has some limitations. First, body mass index usually tends to fluctuate overtime, but most studies used a single measure of BMI at baseline，which may not reflect usual adult weight. Stronger associations between BMI and bladder cancer risk being found in studies that average age of baseline was greater than 50-years old clarified that ages at baseline can affect the association between BMI and risk of bladder cancer. Therefore, future epidemiological studies should consider the cumulative effects of excess body weight on bladder cancer risk. Second, studies included in our meta-analysis used a wide variety of BMI and varying reference categories, which may influence the accuracy of our results to some extent. However, when we restricted the analysis to studies that used standard WHO BMI categories with normal weight (18.50–24.99) as reference category, the results were not substantially changed (S2 Fig). We also addressed that issue by performing a dose-response meta-analysis that was based on comparable percentiles of the distributions of BMI in the underlying studies. Third, it is possible that the observed association between BMI and bladder cancer could be underestimated or overestimated due to unmeasured or residual confounding. Higher level of BMI tends to be associated with other unhealthy behaviors (e.g. lower levels of physical activity, higher alcohol consumption). However, many of the studies in our meta-analysis adjusted for known confounding factors, and the results were consistent in the subgroup analyses.

In summary, findings from this dose-response meta-analysis suggest obesity is associated with linear-increased risk of bladder cancer. Given the rising rates of preobese and obesity worldwide, more in-depth studies are warranted to disentangle the roles of the biological mechanisms involved in obesity-related carcinogenesis.

## Supporting Information

S1 Fig Forest plot of each 5 kg/m^2^ increase in BMI and risk of bladder cancer. Squares indicate study-specific relative risks (size of the square reflects the study-specific statistical weight, i.e., the inverse of the variance); horizontal lines represent 95% CIs; the diamond indicates the summary relative risk estimate with its 95% CI. CI, confidence interval.
(TIF)

S2 Fig Forest plot of BMI and risk of bladder cancer (restricted analysis to studies that using standard WHO body mass index categories). Squares indicate study-specific relative risks (size of the square reflects the study-specific statistical weight, i.e., the inverse of the variance); horizontal lines represent 95% CIs; the diamond indicates the summary relative risk estimate with its 95% CI. CI, confidence interval.
(TIF)

S1 PRISMA Checklist PRISMA 2009 checklist in this meta-analysis. (DOC)

S1 Table Characteristics of prospective studies evaluating body mass index and the risk of bladder cancer. (DOC)
