# Supplementary figures and images for: Obesity and Risk of Bladder Cancer: A Dose-Response Meta-Analysis of 15 Cohort Studies
Source: PLoS One. 2015 Mar 24;10(3):e0119313. doi: 10.1371/journal.pone.0119313 (PMC4372289; doi:10.1371/journal.pone.0119313)

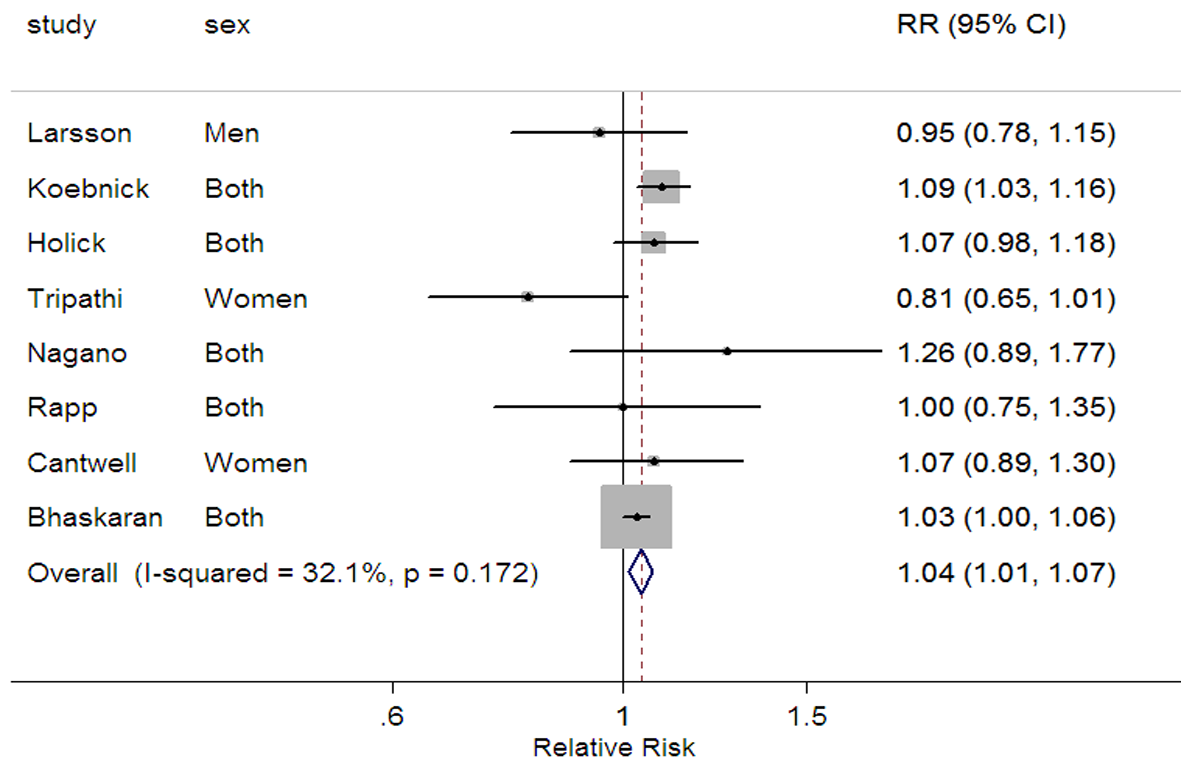

Supplement: S1 Fig — Squares indicate study-specific relative risks (size of the square reflects the study-specific statistical weight, i.e., the inverse of the variance); horizontal lines represent 95% CIs; the diamond indicates the summary relative risk estimate with its 95% CI. CI, confidence interval. (TIF) [file pone.0119313.s001.tif]

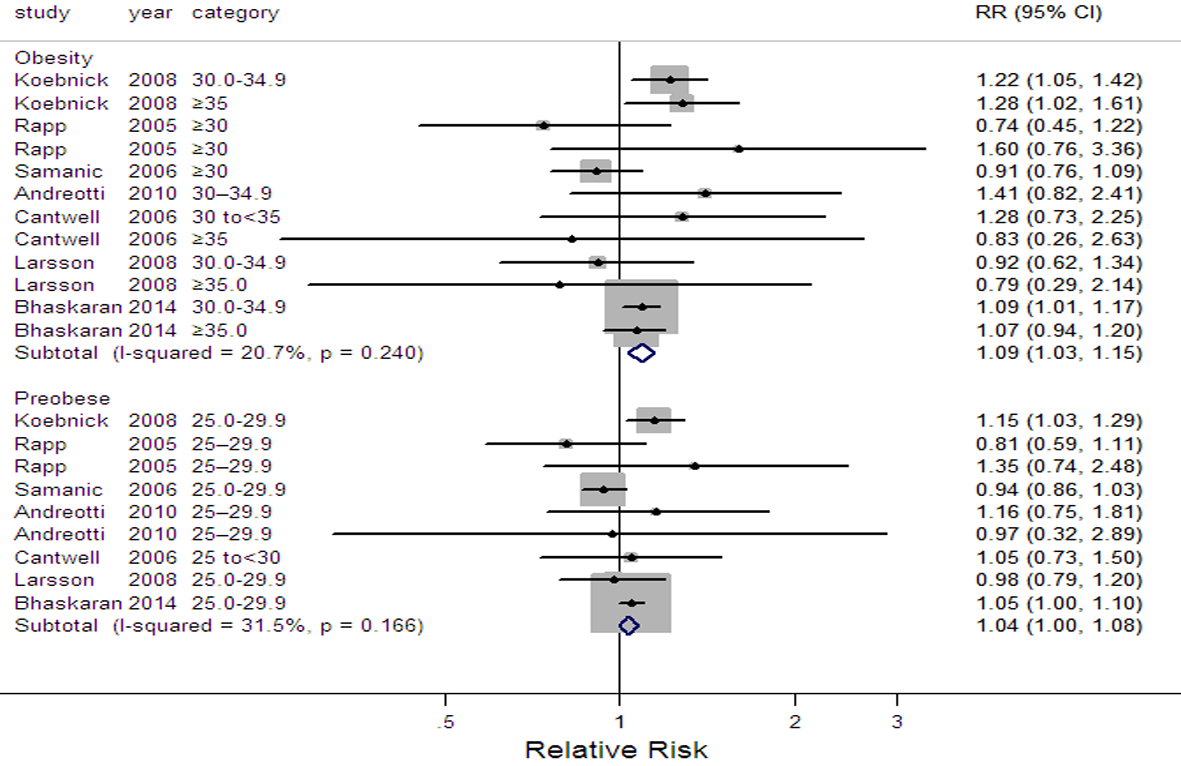

Supplement: S2 Fig — Squares indicate study-specific relative risks (size of the square reflects the study-specific statistical weight, i.e., the inverse of the variance); horizontal lines represent 95% CIs; the diamond indicates the summary relative risk estimate with its 95% CI. CI, confidence interval. (TIF) [file pone.0119313.s002.tif]
