# Supplementary material for: Obesity and Risk of Bladder Cancer: A Dose-Response Meta-Analysis of 15 Cohort Studies
Source: PLoS One. 2015 Mar 24;10(3):e0119313. doi: 10.1371/journal.pone.0119313 (PMC4372289; doi:10.1371/journal.pone.0119313)
Supplement: S1 Table — (DOC) [file pone.0119313.s004.doc]

TableS1. Characteristics of prospective studies evaluating body mass index and risk of bladder cancer

| Study, Year | Country,  follow-up (years) | Cohort size | Sex，Age(Years) | Assessment of BMI | No. cases | No.of person-years | BMI (kg/m^2^) | RR (95%CI) | Adjustment factors |
| --- | --- | --- | --- | --- | --- | --- | --- | --- | --- |
| Larsson et al.  2008 | Swedish, 9.3y | 45906 | M, 60.4y (45-79) | self-report | 176  177  31  4 | 177924  187037  34835  5424 | 18.0-24.9  25.0-29.9  30.0-34.9  ≥35.0 | 1.00(Reference)  0.98(0.79-1.20)  0.92(0.62-1.34)  0.79(0.29-2.14) | age, education, smoking status and pack-years of smoking |
| Koebnick  et al.  2008 | USA, 7.2y | 471760 | M/F:  61.9y (50-71) | self-report | 479  845  301  94 | 1174879  1474882  541963  212917 | 18.5-24.9  25.0-29.9  30.0-34.9  ≥35 | 1.00(Reference)  1.15(1.03-1.29)  1.22(1.05-1.42)  1.28(1.02-1.61) | age, physical activity, gender, smoking status, race/ethnicity, education, marital status, family history of cancer, intakes of red meat, fruit and vegetables, nonalcoholic beverages, and alcohol, menopausal hormone therapy, oral contraceptive use and parity |
| Holick et al.  2007 | USA, M:16y  F:26y | M:47914; F:114621 | M:54.3y  F:42.8y | self-report | M:76  143  147  102  39  F:166  71  52  34  36 | M:124646  197132  188477  127019  56183  F:1467236  556958  335214  253400  235747 | M:18.0-22.9  23.0-24.9  25.0-26.9  27.0-29.9  ≥30.0  F: 18.0-22.9  23.0-24.9  25.0-26.9  27.0-29.9  ≥30.0 | M:1.00(Reference)  1.11(0.84-1.47)  1.14(0.86-1.51)  1.12(0.83-1.51)  1.01(0.68-1.50)  F: 1.00(Reference)  1.04(0.78-1.38)  1.23(0.90-1.69)  1.09(0.75-1.58)  1.31(0.91-1.89) | age, pack-years of cigarette smoking and current smoking |
| Tripathi et al.  2002 | USA,13y | 37459 | F: 55-69 | self-report | 30  27  21  18  16 | 87131  88483  91266  89258  88348 | <=22.89  22.90-25.02  25.04-27.43  27.46-30.67  ≥30.69 | 1.00(Reference)  1.09(0.64-1.84)  0.74(0.41-1.34)  0.69(0.38-1.27)  0.63(0.33-1.19) | age, pack-years of smoking, regular physical activity, diabetes alcohol, married, occupational lifetime |
| Nagano et al.  2000 | Japan, 11.7y | 38540 | M:52.8y  F:56.8y | self-report | 14  37  37  18 | 50326  124848  115235  60882 | <19  19-22  22-25  ≥25 | 1.00(Reference)  1.28(0.70-2.45)  1.50(0.82-2.90)  1.55(0.77-3.21) | age, gender, smoking status, radiation dose, education level, calendar time |
| Samanic et al.  2004 | USA, 12y | White men:  3668486; Black men:832214 | M: 51.3y | measured | 16260  1087  1796  72 | 40665254  2084083  9527566  358756 | White men  <30  ≥30  Black men  <30  ≥30 | White men  1.00(Reference)  1.13(1.06-1.20)  Black men  1.00(Reference)  0.85(0.67-1.08) | age and calendar year |
| Haggstrom  et al.  2011 | Norway,  Austria,  Sweden  11.7y | 578700 | M/F: 44y | NA | M:269  302  316  303  334  F:51  60  70  56  72 | NA | M:21.5±1.3  23.8±0.8  25.4±0.8  27.1±0.9  30.8±2.8  F: 20±1.2  22.3±0.8  24.1±0.8  26.4±1.0  31.7±3.6 | M:1.00(Reference)  1.06(0.89-1.28)  1.06(0.88-1.26)  0.97(0.80-1.16)  1.13(0.94-1.35)  F: 1.00(Reference)  1.00(0.66-1.51)  1.00(0.67-1.50)  0.67(0.44-1.03)  0.87(0.58-1.32) | smoking, five categories of birth year, age at measurement, and stratified for cohort |
| Rapp et al.  2005 | Austria, 9.9y | M:67447  F:78484 | M:41.8y, F:42.5y | measured | M:78  78  19  F:21  22  11 | 330 040  262 144  57173  502 849  208 574  87700 | M:18.5–24.9  25– 29.9  ≥30  F: 18.5 – 24.9  25– 29.9  ≥30 | M:1.00(Reference)  0.81(0.59-1.11)  0.74(0.45-1.22)  F: 1.00(Reference)  1.35(0.74-2.48)  1.60(0.76-3.36) | age at enrolment, smoking status, occupational group |
| Reeves et al.  2007 | UK, 5.4y | 1222630 | F: 55.9y | self-report | 117  149  147  92  110 | NA | <22.5  22.5-24.9  25-27.4  27.5-29.5  ≥30 | 0.99(0.83-1.19)  1.00(Reference)  1.14(0.97-1.34)  1.15(0.93-1.41)  1.07(0.88-1.30) | age, geographical region, socioeconomic status, reproductive history, smoking status, alcohol intake, physical activity, and, where appropriate, time since menopause and use of hormone replacement therapy |
| Samanic et al.  2006 | Swedish, 19y | 362552 | M: 34.3y | measured | 1066  836  128 | NA | 18.5-24.9  25.0-29.9  ≥30 | 1.00(Reference)  0.94(0.86-1.03)  0.91(0.76-1.09) | attained age , and calendar year , and smoking status, and relative to normal weight subjects |
| Jee et al.  2008 | Korea, 10.8y | M:770556 F:443273 | M/F: 46.6y | measured | M:233  743  604  529  19  F:26  99  84  94  8 | NA | M: <20.0  20.0–22.9  23.0–24.9  25.0–29.9  ≥30  F: <20.0  20.0–22.9  23.0–24.9  25.0–29.9  ≥30 | M: 0.84(0.68-1.04)  0.91(0.78-1.05)  1.00(Reference)  1.19(1.01-1.40)  1.02(0.52-1.97)  F: 0.57(0.31-1.03)  0.74(0.49-1.11)  1.00(Reference)  1.10(0.75-1.62)  0.74(0.27-2.06) | age, and smoking status |
| Andreotti et al. 2010 | USA, >10y | M:39628 F:28319 | M/F: 47.7y | self-report | M:0  34  66  28  3  F:1  7  6  2  1 | NA | M: <18.5  18.5–24.9  25–29.9  30–34.9  ≥35  F: <18.5  18.5–24.9  25–29.9  30–34.9  ≥35 | M: .NA  1.00(Reference)  1.16(0.75-1.81)  1.41(0.82-2.41)  .NA  F: NA  1.00(Reference)  0.97(0.32-2.89)  NA  NA | Men: age, smoking status, alcohol drinking; Women: age, exercise; |
| Cantwell et al.  2006 | USA, 15.3y | 54308 | F: 55.4y | measured | 2  103  42  14  3 | 7469  214102  78414  22348  8142 | <18.5  18.5 to<25  25 to<30  30 to<35  ≥35 | 0.55(0.14-2.24)  1.00(Reference)  1.05(0.73-1.50)  1.28(0.73-2.25)  0.83(0.26-2.63) | age, calendar year and smoking status |
| Song et al. 2014 | Finland  20.6y | 54725 | M:44.1yF:44.0y | measured | M:192  F:40 |  | M:18.5-20.9  21.0-22.9  23.0-24.9  25.0-27.4  27.5-29.9  30.0-34.9  ≥35.0  F: 18.5-20.9  21.0-22.9  23.0-24.9  25.0-27.4  27.5-29.9  30.0-34.9  ≥35.0 | M:1.36(0.59-3.12)  1.07(0.59-1.94)  1.00(Reference)  1.48(0.95-2.32)  1.95(1.24-3.09)  1.47(0.85-2.52)  1.91(0.74-4.98)  F:1.88(0.31-11.36)  3.07(0.79-11.89)  1.00(Reference)  2.59(0.71-9.43)  2.69(0.71-10.18)  2.63(0.69-10.00)  1.93(0.32-11.69) | living area, smoking status, leisure-time physical activity, and education at baseline, using age at time scale |
| Bhaskaran et al.2014 | UK  7.5y | 5243978 | M/F:37.9y | measured | 121  2907  3491  1147  310 |  | <18.5  18.5-24.9  25.0-29.9  30.0-34.9  ≥35.0 | 1.22(1.00-1.44)  1.00(Reference)  1.05(1.00-1.10)  1.09(1.01-1.17)  1.07(0.94-1.20) | Sex and age |

Abbreviations: BMI, body mass index; RR, relative risk; CI, confidence interval; NA: not available; M: male; F: female.
